# Ferroelectric-assisted BaTiO_3_/Cr-Zr-HMS (5, 10 & 20) catalysts for efficient visible-light removal of bromocresol green: structure–activity relationship and process optimization

**DOI:** 10.1039/d5ra07033c

**Published:** 2025-12-16

**Authors:** Nastaran Parsafard, Ali Akbar Asgharinezhad, Najmeh Karami

**Affiliations:** a Kosar University of Bojnord, Department of Applied Chemistry North Khorasan Iran n-parsafard@kub.ac.ir +98 58 32427408 +98 58 32258865; b Chemistry and Process Research Department, Niroo Research Institute Tehran Iran

## Abstract

In this study, BaTiO_3_/Cr-Zr-HMS (BTCZH*x*) photocatalysts with different Si/Zr molar ratios were successfully synthesized and evaluated for the visible-light-driven photodegradation of bromocresol green dye. The catalysts were characterized and tested under varying operational parameters, and their performance was optimized using response surface methodology based on central composite design. Three key factors—pH of the dye solution, process time, and Si/Zr molar ratio—were systematically investigated for their influence on the photodegradation efficiency. Statistical analysis confirmed the adequacy of the quadratic model (*R*^2^ = 0.97), with pH identified as the most significant factor, followed by Si/Zr ratio and process time. The optimal conditions (pH = 3, *t* = 35 min, *x* = 20) yielded a maximum PDE of over 95%. The proposed photocatalytic mechanism suggests that under visible-light irradiation, photogenerated electron–hole pairs initiate the formation of highly reactive species (˙OH and ˙O_2_^−^), which synergistically degrade the dye molecules. Furthermore, reusability and stability tests revealed that the BTCZH20 catalyst maintained nearly 90% of its initial activity after five successive cycles, demonstrating excellent structural robustness and practical applicability. These findings highlight the potential of BaTiO_3_/Cr-Zr-HMS composites as efficient and durable photocatalysts for wastewater treatment under visible-light conditions.

## Abstract 2

In this study, BaTiO_3_/Cr-Zr-HMS (BTCZH*x*) photocatalysts with different Si/Zr molar ratios were successfully synthesized and evaluated for the visible-light-driven photodegradation of bromocresol green dye.

## Graphical abstract

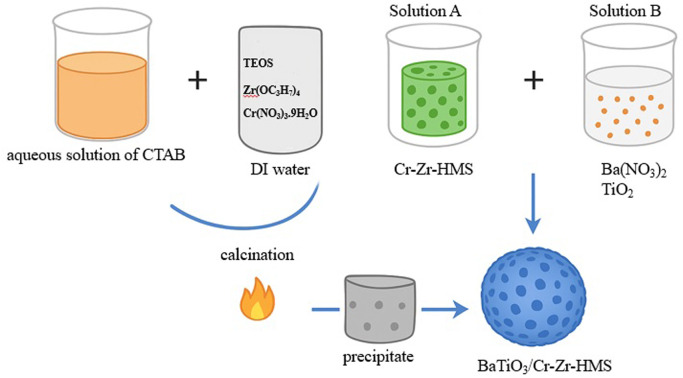

## 1. Introduction

Synthetic dyes are among the most persistent contaminants in aquatic systems due to their chromophoric stability, complex aromatic structures, and resistance to biodegradation. Among these, bromocresol green (BCG)—an anionic triphenylmethane dye widely used as a pH indicator and in textile formulations—exhibits recalcitrant behavior and poses ecotoxicological risks even at low concentrations. Photocatalysis under visible light presents an attractive route for BCG remediation, coupling mild operating conditions with *in situ* generation of reactive oxygen species (ROS) capable of mineralizing dyes to CO_2_, H_2_O, and inorganic ions. However, practical application has been limited by poor visible-light absorption of conventional photocatalysts and rapid recombination of photogenerated charge carriers, challenges that are particularly pronounced for anionic dyes like BCG.^1–4^

Traditional advanced oxidation processes (AOPs), such as Fenton and photo-Fenton reactions, have been extensively used for the degradation of recalcitrant dyes and organic pollutants.^5,6^ These methods can achieve relatively high pollutant abatement; however, they also suffer from several drawbacks, including the requirement for large amounts of H_2_O_2_ and Fe^2+^ reagents, the generation of secondary iron-rich sludge, narrow operational pH windows (typically pH ≈ 2–4), and limited efficiency under visible-light irradiation. Such limitations reduce their applicability in sustainable wastewater treatment.^5,6^

In contrast, visible-light-driven photocatalytic systems—particularly ferroelectric-assisted materials such as BaTiO_3_-based catalysts—offer improved charge separation, higher stability, and reduced environmental impacts.^7^ Therefore, developing hybrid systems such as BaTiO_3_/Cr-Zr-HMS can provide an efficient and greener alternative to classic AOPs for treating recalcitrant dyes like BCG.

Previous studies have explored various approaches for the removal of BCG from aqueous solutions, including advanced oxidation processes (AOPs), semiconductor photocatalysis, and adsorption on biochar materials. Table 1 summarizes key studies, their methodologies, main findings, and limitations.

**Table 1:** Overview of previous studies on BCG removal

Method/catalyst	Key findings/efficiency	Limitations/remarks
UV/Co^2+^/peroxymonosulfate^1^	Effective BCG degradation *via* advanced oxidation; mechanistic insight provided	Requires additional chemical reagents; possible secondary byproducts
Reduced ZnO photocatalysis^2^	Comparative study of cationic/anionic dyes; kinetic modeling	Limited visible-light absorption; low stability in repeated cycles
Cucumber straw biochar adsorption^3^	High adsorption capacity for BCG from aqueous solution	Only adsorption; no degradation, potential desorption
Pine cone-derived activated biochar^4^	Efficient BCG removal; modeling supported	Adsorption only, not mineralization; regeneration issues
Zr-doped TiO_2_ (ref. 30)	Enhanced photocatalytic degradation of bisphenol A	No study on BCG specifically; limited visible-light activity
Semiconductor photocatalysis^31^	pH significantly affects photocatalytic efficiency	Mechanistic details for BCG not included
Ag/BaTiO_3_ aerogel^21^	High photodegradation efficiency; visible-light active	Expensive noble metal usage; scalability issues

These studies demonstrate that while several techniques can achieve BCG removal, they suffer from limitations such as restricted visible-light activity, reliance on additional reagents, incomplete mineralization, or limited stability. Consequently, developing a hybrid photocatalyst that integrates ferroelectric-assisted charge separation, visible-light activity, and tunable surface acidity remains a significant challenge, motivating the current work.

To address these limitations, we developed a BaTiO_3_/Cr-Zr-HMS composite catalyst that integrates three synergistic elements. First, ferroelectric BaTiO_3_ (BTO) provides spontaneous polarization, creating an internal electric field that drives directional separation of photoexcited electrons and holes, mitigating recombination and enhancing surface redox reactions.^8–11^ Second, Cr-Zr-modified hexagonal mesoporous silica (HMS) serves as a high-surface-area scaffold, offering mesoporosity for efficient mass transport and tunable acidity to optimize adsorption of anionic BCG.^12–15^ Chromium centers act as visible-light chromophores, participating in ligand-to-metal charge transfer (LMCT) and interfacial redox processes, while zirconium incorporation improves hydrothermal stability and anchoring of active sites. Third, systematic variation of the Si/Zr ratio (5, 10 & 20) allows fine-tuning of surface acid–base properties, point of zero charge (PZC), and interfacial electronic structure, directly influencing adsorption-reaction coupling and catalytic efficiency.^16–18^

The resulting BaTiO_3_/Cr-Zr-HMS catalysts are designed to efficiently harvest visible light, route charge carriers away from recombination through ferroelectric polarization and junction band alignment, pre-adsorb BCG at the solid–liquid interface, and maintain rapid intra-pore mass transport. Under visible irradiation, degradation can proceed *via* three complementary pathways: direct photocatalysis, dye-sensitized electron injection, and LMCT-assisted oxidation at surface Cr–O and Zr–O sites. Importantly, the ferroelectric-driven charge separation enhances ROS generation without requiring plasmonic or defect-state assistance, while the Si/Zr ratio governs selective dye uptake and interfacial reaction kinetics.^19–21^

This study presents a comprehensive investigation of BaTiO_3_/Cr-Zr-HMS composites for visible-light BCG degradation, encompassing materials synthesis, structural and optical characterization, reactor-level performance testing, stability and reusability evaluation, and response surface methodology (RSM)-guided optimization of key operational parameters. By combining ferroelectric-assisted charge separation with visible-active, acid-tunable mesoporous scaffolds and systematic process optimization, this work establishes a materials-to-process blueprint for practical water treatment applications targeting recalcitrant anionic dyes like BCG—a strategy not previously reported in the literature.

## 2. Materials and methods

### 2.1. Catalyst preparation

Cr-Zr-modified hexagonal mesoporous silica (Cr-Zr-HMS) with different Si/Zr molar ratios (5, 10, and 20) was synthesized following a modified sol–gel method as reported in our previous work.^14^ Briefly, an aqueous solution of hexadecyltrimethylammonium bromide (CTAB, 0.03 mol) was prepared in deionized water under continuous stirring at 40 °C. To this solution, a mixture of tetraethyl orthosilicate (TEOS) as the silica source, zirconium (iv) *n*-propoxide (Zr(OC_3_H_7_)_4_) as the zirconium source, and chromium (iii) nitrate nonahydrate (Cr(NO_3_)_3_·9H_2_O) as the chromium source was added dropwise while maintaining vigorous stirring. The molar composition of the gel was adjusted to achieve the desired Si/Zr ratios of 5, 10, and 20, with a fixed Cr loading amount. The pH of the reaction mixture was adjusted to ∼10 using 1.0 M NaOH solution to promote hydrolysis and condensation. The mixture was aged at ambient temperature for 24 h, followed by filtration, thorough washing with deionized water, and drying at 100 °C overnight. The dried solids were calcined in air at 550 °C for 6 h to remove the surfactant template, yielding the mesoporous Cr-Zr-HMS supports.

**Scheme 1:**
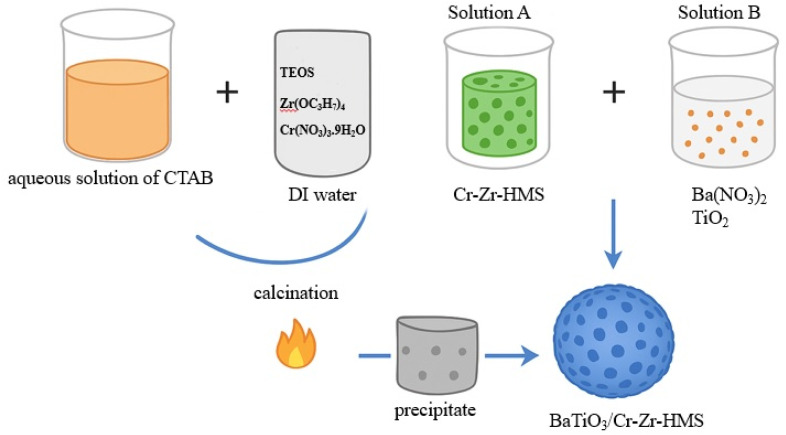
Schematic illustration of the synthesis process of BaTiO_3_/Cr-Zr-HMS composites. The precipitate was separated by centrifugation at 3000 rpm for 10 min, washed thoroughly with deionized water, and dried at 110 °C for 2 h. The dried material was calcined in a muffle furnace under the stepwise heating program (Schematic 1). The same procedure was applied for the preparation of BaTiO_3_/Cr-Zr-HMS(10) and BaTiO_3_/Cr-Zr-HMS(20), denoted as BTCZH10 and BTCZH20, respectively.

BaTiO_3_/Cr-Zr-HMS composites were synthesized *via* an *in situ* precipitation method. For a representative synthesis of BaTiO_3_/Cr-Zr-HMS(5) (BTCZH5), 1.0 g of Cr-Zr-HMS(5) was dispersed in an appropriate volume of deionized water (solution A) under magnetic stirring. In a separate beaker (solution B), 0.02 mol of barium nitrate [Ba(NO_3_)_2_] and 0.02 mol of titanium dioxide (TiO_2_) were dispersed in 100 mL of deionized water. The pH of Solution B was adjusted to the range of 6–8 using 0.025 M aqueous ammonia (NH_4_OH). Solution B was then slowly added to solution A under constant stirring, and the resulting suspension was stirred for an additional 2 h. The mixture was then allowed to stand undisturbed at room temperature for 24 h to facilitate precipitation and crystallization (Scheme 1).

### 2.2. The characterization of BaTiO_3_/Cr-Zr-HMS composite catalyst

The comprehensive characterization of the BaTiO_3_/Cr-Zr-HMS catalysts with varying Si/Zr ratios (5, 10, and 20) was performed using a suite of complementary analytical techniques to elucidate their structural, optical, morphological, and surface properties in detail. Structural phase identification and crystallinity were examined by X-ray diffraction (XRD) using a Philips PW1730 diffractometer equipped with Cu-Kα radiation (*λ* = 1.5406 Å), enabling determination of phase composition, BaTiO_3_ ferroelectric domain retention, and mesostructural order within the HMS framework. Molecular bonding and surface functional groups were analyzed by Fourier Transform Infrared Spectroscopy (FTIR) using a Nicolet™ iS™ 10 spectrometer in the 400–4000 cm^−1^ range, allowing detection of Si–O–Si framework vibrations, Zr–O and Cr–O linkages, and hydroxyl-related surface functionalities. Optical absorption characteristics were investigated by UV-Vis diffuse reflectance spectroscopy (UV-Vis/DRS) on an Evolution 300 spectrophotometer to determine visible-light harvesting capability, identify ligand-to-metal charge transfer (LMCT) features, and estimate the optical band gap energies *via* Kubelka–Munk analysis. Surface morphology and elemental distribution were examined using a SEM VEGA3 scanning electron microscope, providing high-resolution micrographs of particle size, mesopore organization, and BaTiO_3_ dispersion within the silica matrix.

Transmission electron microscopy (TEM) characterization was carried out using a ZEISS EM10 transmission electron microscope. For sample preparation, the catalyst powders were ultrasonically dispersed in ethanol, and a few drops of the suspension were deposited onto carbon-coated copper grids. The grids were then allowed to dry naturally at room temperature. TEM images were recorded at an accelerating voltage of 80–100 kV to visualize the morphological and textural feature of the synthesized material.

Elemental composition and metal loading were quantitatively confirmed through X-ray fluorescence (XRF) analysis using an XRF-8410 Rh 60 kV instrument, ensuring accurate assessment of Ba, Ti, Cr, and Zr incorporation. Textural properties, including specific surface area, total pore volume, and pore size distribution, were determined from N_2_ adsorption–desorption isotherms measured on a BET BELSORP Mini II apparatus, with calculations performed *via* the Brunauer–Emmett–Teller (BET) and Barrett–Joyner–Halenda (BJH) methods to reveal mesoporosity preservation after BaTiO_3_ and metal incorporation. This multi-technique approach provided a holistic understanding of the interplay between structure, surface chemistry, and photocatalytic performance in the BaTiO_3_/Cr-Zr-HMS system.

### 2.3. Evaluating photocatalytic activity

The photocatalytic performance of the BaTiO_3_/Cr-Zr-HMS catalysts (Si/Zr ratios of 5, 10, and 20) was evaluated in a custom-designed batch reactor equipped with precise temperature regulation. The system employed a double-walled quartz reaction cell integrated with a circulating water jacket (bain-marie cooling) connected to a thermostat, in combination with an internal ventilation setup comprising strategically placed cooling fans inside the light-proof chamber. This configuration ensured that the reaction temperature was maintained at 25 ± 1 °C during visible-light irradiation (300 W Xenon lamp), minimizing thermal artifacts from the light source. For each experiment, 60 mL of aqueous Bromocresol Green (BCG) solution of known initial concentration was mixed with 0.25 g of catalyst. The solution pH was adjusted to the desired value (ranging from acidic pH 3 to alkaline pH 11) using calibrated volumes of 0.1 M HCl or NaOH. Prior to illumination, the suspension was magnetically stirred in the dark for 60 min to establish adsorption–desorption equilibrium between the dye molecules and catalyst surface. Visible-light irradiation was provided by a calibrated lamp system, and degradation experiments were performed for up to 35 min, with aliquots collected every 5 min. Collected samples were immediately centrifuged at 3000 rpm for 10 min to separate the solid catalyst from the liquid phase. The residual concentration of BCG in the supernatant was quantified using a UV-Vis spectrophotometer (evolution 300) by measuring the absorbance at the dye's major absorption wavelength (*λ*_max_ ≈ 618 nm). Although the intensity of this peak varies slightly with pH, it provides a reliable measure for monitoring dye concentration under the tested conditions.

**Fig. 1:**
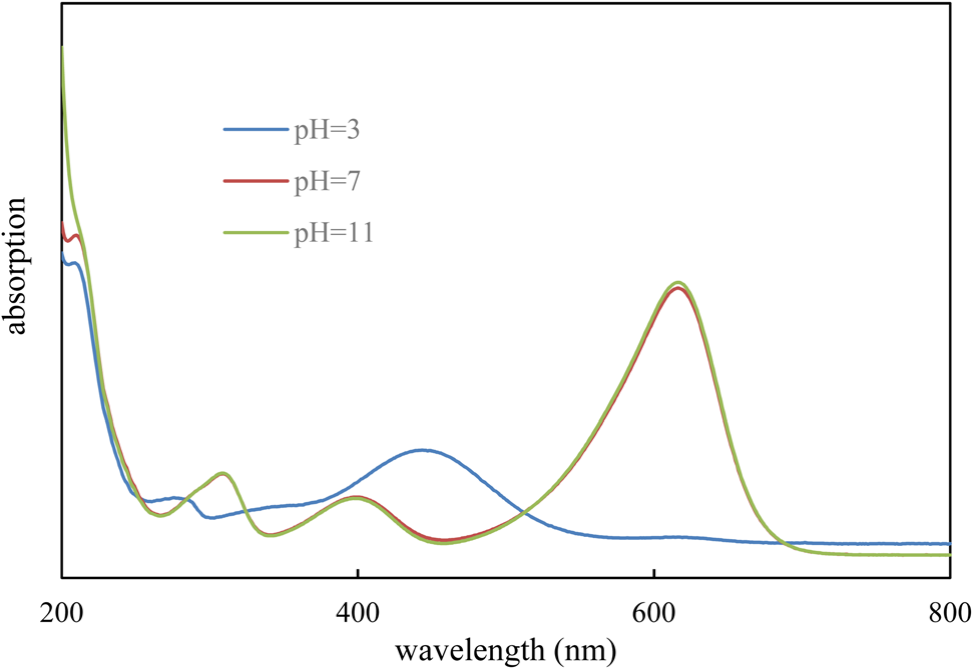
UV-Vis absorption spectrum of the BCG at different pH values.

The UV-Vis absorption spectrum of the dye was recorded at different pH values to identify the main absorption bands. The spectrum exhibits three prominent peaks at approximately 308, 406, and 618 nm. The 618 nm peak corresponds to the primary π → π* transition of the conjugated system, while the peaks at 308 and 406 nm are attributed to additional π → π* and *n* → π* transitions, respectively. The relative intensity of the 618 nm band varies with pH: it decreases under acidic conditions due to protonation of the chromophore and increases under basic conditions owing to enhanced conjugation, although its position remains largely unchanged. These results indicate that 618 nm is generally suitable for monitoring photocatalytic degradation, while the other electronic transitions contribute to overall light absorption under varying conditions. Similar spectral features and assignments have been reported in the literature for azo dyes^22^ (Fig. 1).

The photodecolorization efficiency (PDE) was calculated using the following expression:1
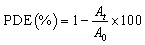
where *A*_0_ is the absorbance of the initial concentration of BCG and *A*_*t*_ is the absorbance of BCG concentration at different times under visible light.

### 2.4. Stability and reusability test

The long-term stability and reusability of the BaTiO_3_/Cr-Zr-HMS photocatalysts were assessed under the optimized reaction conditions obtained from the RSM analysis. Photocatalytic degradation of BCG was performed at pH 3, initial dye concentration of 10 ppm, reaction temperature of 25 ± 1 °C, and catalyst dosage of 0.25 g dispersed in 60 mL aqueous solution under visible-light irradiation. Visible-light irradiation was provided by a 300 W Xenon lamp equipped with a visible-light cut-off filter (*λ* > 420 nm), ensuring illumination predominantly in the visible range (400–700 nm). Each experimental run was conducted for 35 min, and the PDE was calculated from the absorbance at 618 nm. After completion of each run, the catalyst was recovered from the suspension by centrifugation at 3000 rpm for 10 min. The separated solid was thoroughly washed twice with deionized water and once with ethanol to remove any adsorbed organic residues. The washed catalyst was then dried at 110 °C for 12 h before reuse. This recovery and reuse procedure was repeated for five consecutive cycles under identical operational conditions. The stability of the photocatalyst was evaluated by comparing the PDE values across cycles.

### 2.5. Experimental design and optimization by RSM

Response surface methodology (RSM) coupled with central composite design (CCD) was employed to optimize the photocatalytic degradation of BCG. The key factors investigated were solution pH, reaction time, and Si/Zr molar ratio, which significantly influence photodegradation efficiency (PDE). A total of 20 strategically designed experiments were performed, covering the full range of these variables. PDE was measured under visible-light irradiation, and the data were analyzed to construct a response surface model. This model enabled prediction of PDE across the experimental domain and identification of optimal conditions for maximum photocatalytic efficiency.

## 3. Results and discussion

### 3.1. Characterization of the synthesized catalysts

**Fig. 2:**
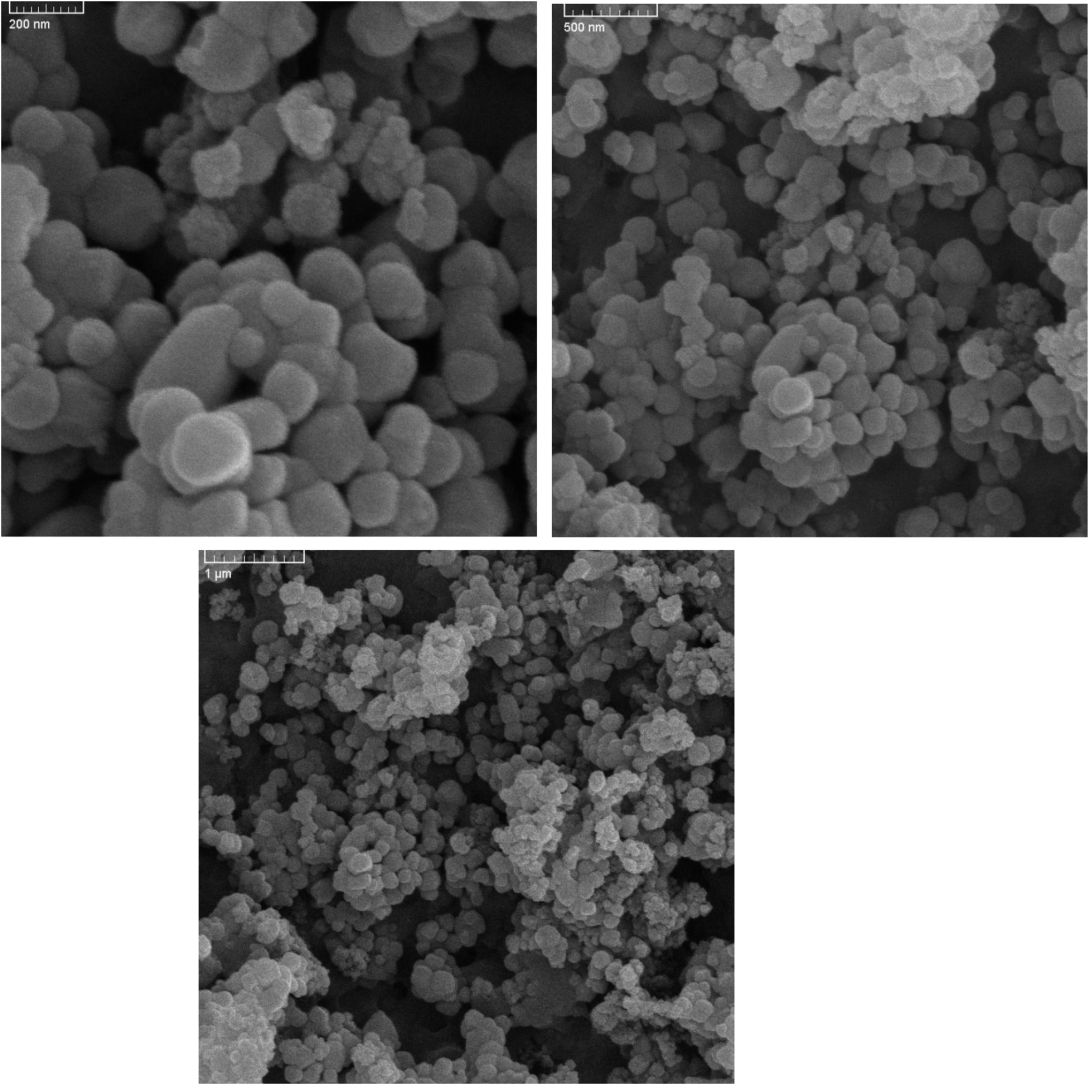
SEM micrographs of BTCZH*x* catalyst at different magnifications as 200 nm, 500 nm, and 1 µm.

SEM image (Fig. 2) revealed that the BaTiO_3_/Cr-Zr-HMS catalyst exhibits a highly ordered spherical morphology, with particles closely packed in a uniform arrangement. The observed spheres possess smooth surfaces and consistent size distribution, suggesting a controlled nucleation-growth process during synthesis. The regular packing and absence of significant agglomeration indicate that the synthesis route effectively maintained particle dispersion while promoting structural uniformity. This ordered arrangement can enhance the photocatalytic performance by maximizing the accessible surface area, facilitating light scattering within the catalyst layer, and promoting efficient mass transport of reactants and products through the mesoporous framework. The uniform spherical morphology is consistent with the templated growth mechanism of HMS, while the incorporation of BaTiO_3_ and Cr-Zr oxides appears to have preserved the mesostructural order without inducing collapse or severe distortion of the framework.

**Fig. 3:**
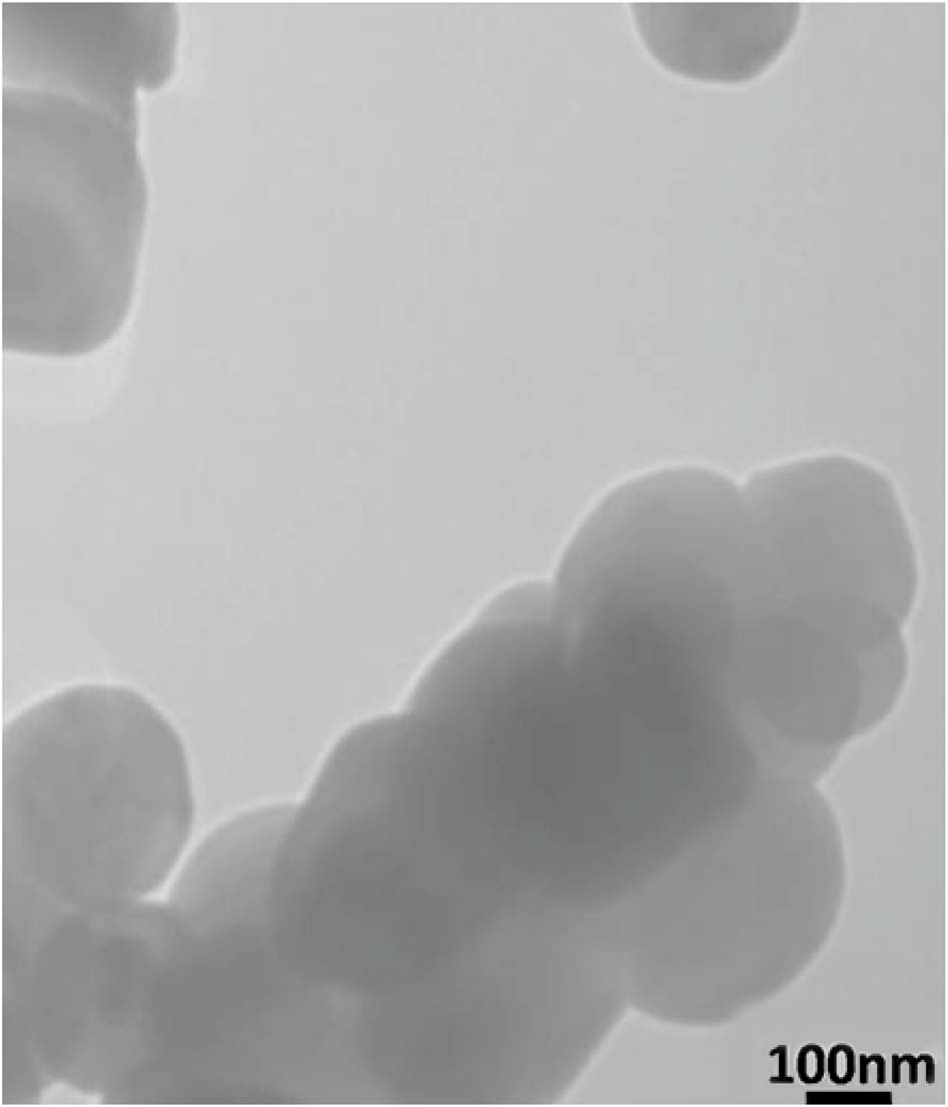
TEM image of the synthesized BaTiO_3_/Cr-Zr-HMS composite.

TEM image (Fig. 3) of the BaTiO_3_/Cr-Zr-HMS composites show that the materials consist of interconnected spherical nanoparticles forming cluster-like mesoporous domains. The contrast distribution indicates uniform dispersion of BaTiO_3_ within the Cr-Zr-HMS framework. The morphology observed is consistent with the expected HMS-type mesoporous structure and is in good agreement with the SEM results. No large crystalline agglomerates were detected, confirming successful integration of BaTiO_3_ into the mesoporous matrix.

**Fig. 4:**
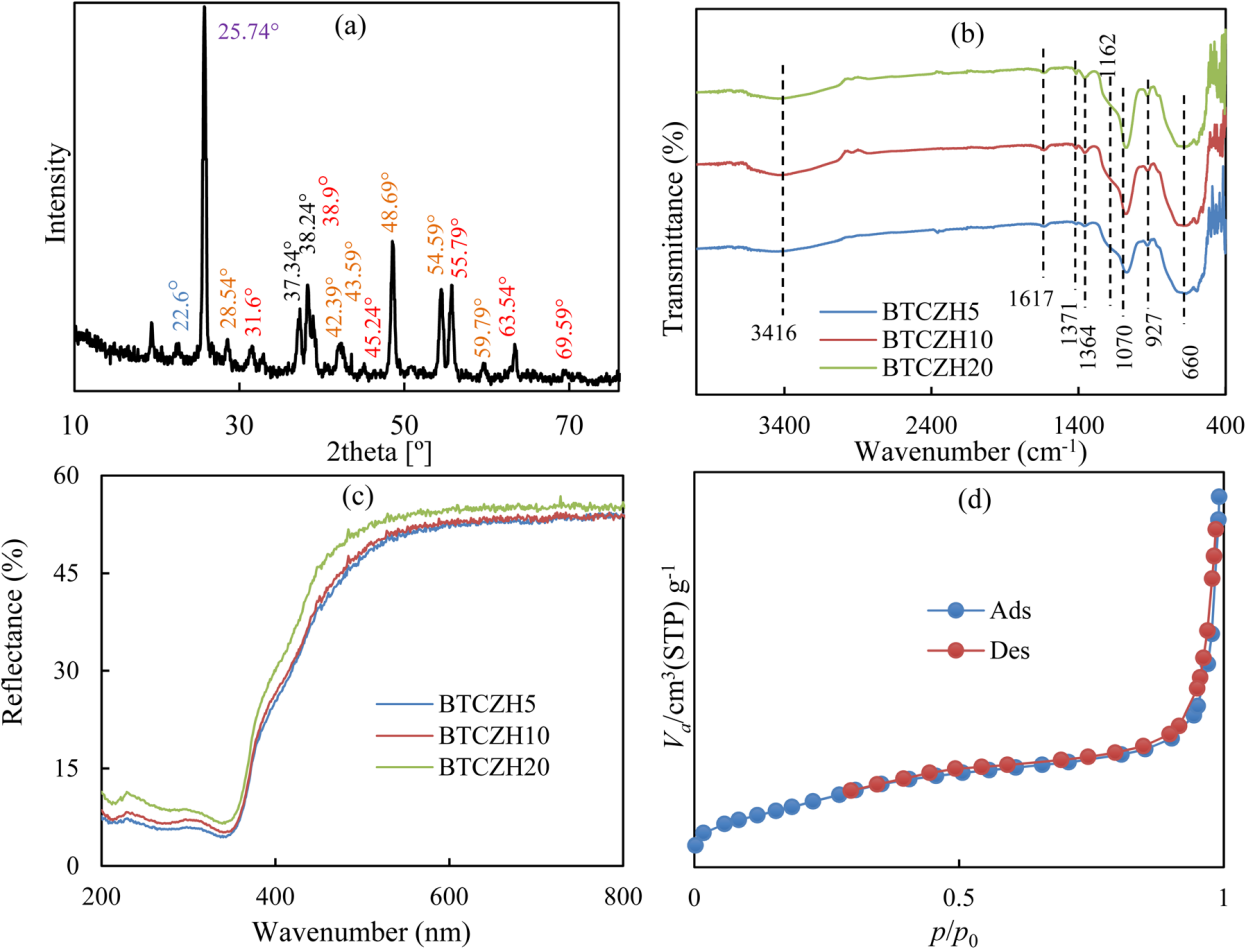
(a) The XRD pattern, (b) the FTIR spectrum, (c) the UV-vis DRS spectrum and (d) BET isotherm of BTCZH*x* catalyst.

The XRD (Fig. 4a) pattern of the synthesized BTCZH*x* composite reveals a combination of crystalline and amorphous features, indicating the successful incorporation of the perovskite phase within the mesoporous silica framework. A broad, low-intensity halo centered at approximately 2*θ* ≈ 22.6° is attributed to the amorphous silica walls of the HMS support, a characteristic feature of mesoporous siliceous materials that confirms preservation of the ordered mesoporous structure after incorporation of the active components.^23,24^ Superimposed on this amorphous background are sharp reflections corresponding to crystalline BaTiO_3_, demonstrating that the perovskite phase was effectively formed under the applied synthesis conditions. The major BaTiO_3_ reflections are observed at 2*θ* ≈ 31.64° (110), 38.94° (111), 45.24° (200)/(002), 55.79° (211), 63.54° (220)/(202), and 69.59° (310)/(212), which are consistent with standard JCPDS data for tetragonal or cubic BaTiO_3_.^25,26^ The presence of closely spaced peaks around 45° suggests partial peak splitting, which may be indicative of weak tetragonality; however, the relatively low calcination temperature (≤300 °C) likely led to poorly developed crystallinity, favoring a pseudo-cubic structure. The formation of crystalline BaTiO_3_ at such mild conditions highlights the potential of the adopted synthesis route to integrate functional perovskite phases into thermally sensitive mesoporous supports. In addition to BaTiO_3_, several peaks suggest the presence of secondary phases. A prominent reflection at 25.74° is in close agreement with the (101) plane of anatase TiO_2_ (JCPDS 21-1272), implying incomplete reaction of Ti precursors or retention of anatase crystallites after calcination.^27,28^ Furthermore, reflections at 28.54°, 42.39°, 43.59°, 48.69°, 54.59°, and 59.79° correspond to orthorhombic BaCO_3_ (witherite, JCPDS 05-0378), a phase commonly observed in low-temperature BaTiO_3_ syntheses due to stabilization of Ba species as carbonates.^29^ The incomplete decomposition of BaCO_3_ is consistent with the modest calcination temperature and could be minimized by higher-temperature treatment under CO_2_-controlled or CO_2_-free conditions. Minor peaks at ∼37–38° and 54–55° may also contain overlapping contributions from both anatase and BaTiO_3_ reflections. Interestingly, characteristic peaks of crystalline ZrO_2_ are absent, suggesting that Zr species are predominantly incorporated into the Si–O–Zr network of HMS rather than forming separate ZrO_2_ domains. Similarly, diffraction peaks from crystalline Cr_2_O_3_ are not distinct, which may be due to the high dispersion of Cr species within the silica framework or incorporation into the perovskite lattice at low concentrations. This high dispersion is advantageous for catalytic applications, as it enhances the availability of active sites and reduces electron–hole recombination in photocatalytic processes.

The FTIR spectra (Fig. 4b) of the synthesized BaTiO_3_/Cr-Zr-HMS catalysts display characteristic vibrational bands corresponding to both the perovskite phase and the mesoporous silica support. The broad and intense band observed around 3416 cm^−1^ is attributed to the O–H stretching vibrations of surface hydroxyl groups and adsorbed water molecules on the catalyst surface, indicating the presence of hydrophilic sites in the mesoporous HMS matrix.^22^ The band at 1617 cm^−1^ corresponds to H–O–H bending vibrations of physically adsorbed water, confirming the retention of water molecules in the pores of the HMS structure.^22^ Weak bands at 1371 and 1364 cm^−1^ can be assigned to the stretching vibrations of carbonate species (CO_3_^2−^), likely arising from the incomplete decomposition of BaCO_3_ during the mild calcination process.^22^

In the fingerprint region, the intense bands observed at 1162 and 1070 cm^−1^ are associated with Si–O–Si asymmetric stretching vibrations, characteristic of the mesoporous silica network.^23^ The bands around 927 and 660 cm^−1^ are assigned to Ti–O and Ba–O stretching vibrations of the BaTiO_3_ perovskite lattice, confirming the formation of the perovskite phase within the silica framework.^8,9^ Additional peaks at 595, 467, and 421 cm^−1^ are consistent with Ti–O–Ti bending vibrations and metal–oxygen lattice modes, indicative of the incorporation of Cr and Zr species into the HMS framework and their possible interaction with BaTiO_3_.^8,24^ A small shoulder at 409 cm^−1^ may correspond to Zr–O vibrations in the amorphous Cr-Zr-HMS support, reflecting the high dispersion of Zr species within the silica network without formation of separate crystalline ZrO_2_ domains.^24^

Overall, the FTIR spectra confirm the coexistence of BaTiO3 perovskite, residual carbonate, and a mesoporous siliceous framework with incorporated Cr and Zr species. The consistency of the vibrational features among the three catalysts suggests that varying the Si/Zr ratio does not significantly alter the primary functional groups or the overall structural framework, although subtle differences in metal–oxygen band intensities could influence catalytic activity.

The UV-Vis DRS (Fig. 4c) spectrum of the BTCZH5 catalyst demonstrates strong absorption in the UV region and relatively low reflectance across the measured wavelengths. In the 200–326 nm range, reflectance varies between ∼5–7.6%, indicating efficient photon absorption due to electronic transitions from the valence band (VB) to the conduction band (CB) typical of wide-bandgap semiconductors like BaTiO_3_ doped with Cr and Zr. The gradual decrease in reflectance up to ∼270 nm highlights strong absorption of high-energy UV photons, while the relatively stable reflectance at longer wavelengths suggests limited visible-light absorption, consistent with Cr-Zr doped BaTiO_3_. The absorption edge of BTCZH5 is observed around 370–380 nm, corresponding to an estimated optical bandgap of approximately 3.25 eV (*E* = 1240/*λ*, with *λ* in nm). Reflectance gradually increases from ∼5% at 327 nm to ∼40% at 454 nm and further to ∼52–55% in the 583–970 nm range, indicating a progressive decrease in light absorption from near-UV to near-infrared (NIR) wavelengths. Minor reflectance fluctuations are attributed to nanoscale surface heterogeneity and light scattering. The UV-Vis DRS analysis of BTCZH10 and BTCZH20 catalysts revealed spectral features closely resembling those of BTCZH5, indicating that Cr-Zr-HMS loading does not significantly alter the fundamental optical response of the BaTiO_3_ matrix. All three samples exhibit strong UV absorption with low reflectance in the 200–380 nm range, consistent with direct electronic transitions from the valence band to the conduction band in wide-bandgap semiconductors. The absorption edges for BTCZH5, BTCZH10, and BTCZH20 were observed at ∼380 nm, corresponding to estimated optical bandgap energies of approximately 3.25, 3.26, and 3.27 eV, respectively. The slight increase in bandgap with higher HMS content may be attributed to subtle structural modifications and reduced defect states at the BaTiO_3_ surface, which can limit mid–gap transitions. In the visible region (400–800 nm), all catalysts show similar reflectance trends, confirming that the photocatalytic performance differences among these samples are not primarily governed by their optical bandgap values.

The textural properties of the synthesized BaTiO_3_/Cr-Zr-HMS catalysts with different Si/Zr ratios were evaluated by N_2_ adsorption–desorption measurements at 77 K. The adsorption–desorption isotherms of BTCZH5 (Fig. 4d) display a typical type IV behavior with a distinct hysteresis loop, indicative of mesoporous structures. The BET surface area (*S*_BET_) for BTCZH5 was determined to be 248.0 m^2^ g^−1^, with a total pore volume (*V*_p_) of 0.531 cm^3^ g^−1^ and an average pore diameter (*d*_P_) of 8.56 nm. The Langmuir surface area and BJH analysis further confirmed the mesoporosity and uniform pore distribution. Increasing the Si/Zr ratio led to a progressive enhancement in the textural properties. BTCZH10 exhibited a higher BET surface area of 312.5 m^2^ g^−1^, a pore volume of 0.612 cm^3^ g^−1^, and a slightly reduced pore diameter of 8.02 nm, indicating a denser packing of mesopores. Similarly, BTCZH20 demonstrated the highest BET surface area (372.0 m^2^ g^−1^) and pore volume (0.689 cm^3^ g^−1^), with a mean pore diameter of 7.66 nm (Fig. S2). These trends (Table 2) suggest that increasing the silica content leads to an improved mesoporous network with higher surface area and pore volume, while slightly narrowing the pore channels.

**Table 2:** Textural and surface properties of the prepared catalysts

Catalysts	*S*_BET_ (m^2^ g^−1^)	*V*_p_ (cm^3^ g^−1^)	*d*_P_ (nm)	Si/Zr
BTCZH5	248.0	0.531	8.56	4.9
BTCZH10	312.5	0.612	8.02	9.4
BTCZH20	372.0	0.689	7.66	18.5

The hysteresis loops observed for all catalysts are consistent with type H1 according to IUPAC classification, reflecting well-defined cylindrical mesopores. The enhancement of surface area and pore volume with increasing Si/Zr ratio can be attributed to the higher silica content providing a more extensive framework for Cr-Zr incorporation, which may favor adsorption sites and increase the accessibility of reactants in catalytic processes. Overall, these textural properties indicate that the prepared catalysts possess suitable mesoporosity and high surface area, which are beneficial for photocatalytic applications.

### 3.2. Performance of BTCZH*x* composite catalyst for the BCG degradation

**Fig. 5:**
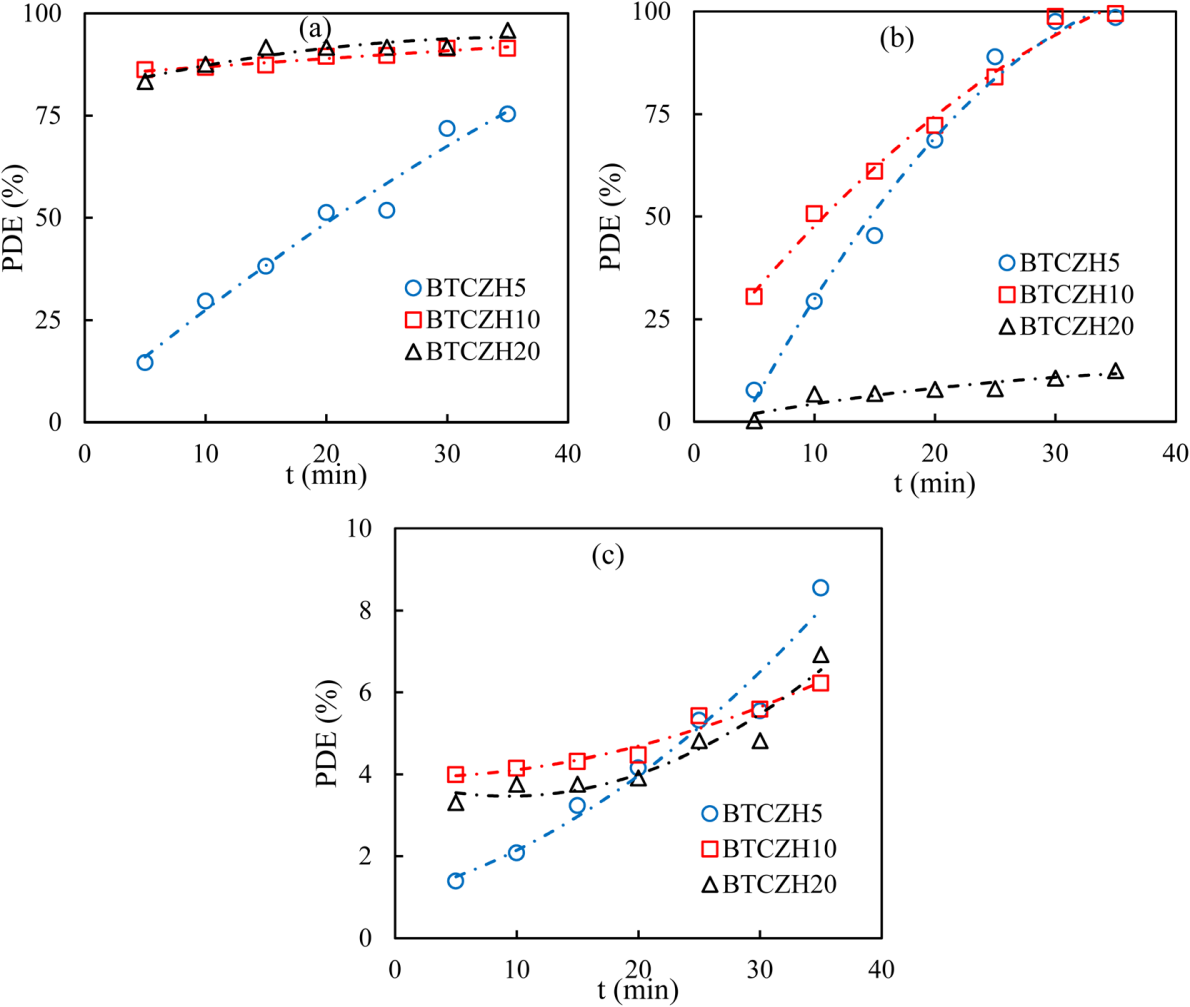
PDE of BCG dye as a function of process time for BTCZH*x* catalysts at (a) pH = 3, (b) pH = 7, and (c) pH = 11 under visible-light irradiation at ambient condition.

The photocatalytic performance of the BTCZH*x* catalysts (Fig. 5) with different Si/Zr ratios (*x* = 5, 10, and 20) showed a strong dependence on solution pH, directly linked to surface charge behavior, adsorption affinity for the anionic dye BCG, and the intrinsic charge separation capacity of the ferroelectric-mesoporous hybrid structure.

It is noted that all photocatalytic experiments were performed in triplicate to ensure reproducibility. For each catalyst and at each pH and irradiation time point, the degradation efficiency was calculated as the mean value of three independent runs. The corresponding standard deviations (SD) are reported in Table S1 in the SI. The trends observed in the degradation curves remained consistent over all repeated experiments, confirming the reliability of the measured values.

At pH 3, all three catalysts achieved high degradation efficiencies (>91% for BTCZH10 and BTCZH20, 75% for BTCZH5 at 35 min). Under acidic conditions, surface protonation of silanol groups and Zr-OH species renders the catalyst surface positively charged, promoting electrostatic attraction with the anionic dye and enhancing the pre-adsorption step, which is critical for efficient interfacial electron transfer. The higher initial rates for BTCZH10 (*k* ≈ 0.17 min^−1^) and BTCZH20 (*k* ≈ 0.19 min^−1^) suggest that a balanced or lower Zr content supports rapid dye oxidation in strongly acidic environments, while BTCZH5 exhibits slower initial kinetics (*k* ≈ 0.033 min^−1^), possibly due to overly strong adsorption leading to partial site blockage or less efficient light penetration.

At pH 7, BTCZH10 demonstrated the best performance (≈99.5% degradation in 35 min, *k* ≈ 0.066 min^−1^), followed closely by BTCZH5 (≈98.5%, *k* ≈ 0.037 min^−1^). In neutral medium, the surface charge approaches the point of zero charge (PZC) for the Zr-modified mesoporous silica, and a moderate Zr content (Si/Zr = 10) appears optimal for achieving a balance between sufficient surface acidity, effective adsorption of BCG, and minimal recombination losses. By contrast, BTCZH20 (low Zr content, Si/Zr = 20) displayed poor performance (≈12.5% degradation, *k* ≈ 0.005 min^−1^) due to a more negative surface potential, which repels the anionic dye, resulting in limited interfacial interactions and reduced reactive oxygen species (ROS) generation efficiency.

In alkaline conditions (pH 11), all catalysts exhibited markedly reduced activity (<9% degradation, *k* ≈ 0.002–0.003 min^−1^). The negative surface charge in basic medium leads to strong electrostatic repulsion with the anionic dye, while excess OH^−^ ions can act as scavengers for photogenerated holes (h^+^) and hydroxyl radicals (˙OH), suppressing the main oxidation pathways. This performance drop indicates that both adsorption and ROS-mediated pathways are strongly inhibited in high-pH environments.

The observed trends can be explained by the synergistic roles of the catalyst components. The ferroelectric BaTiO_3_ phase generates an internal electric field that facilitates directional charge separation and suppresses electron–hole recombination. Cr^3+^ dopants extend visible-light absorption *via* ligand-to-metal charge transfer (LMCT) and d-d transitions, while also acting as redox-active centers for O_2_ and H_2_O activation. Zr incorporation into the HMS framework enhances surface acidity, improves thermal and hydrothermal stability, and shifts the PZC toward higher values, thereby tuning the electrostatic interaction with the dye. The mesoporous HMS scaffold provides large pore volume and accessibility, enabling effective diffusion of the relatively bulky BCG molecules to active sites. Among the tested ratios, Si/Zr = 10 delivers the optimal compromise between acidity, charge separation, and mesostructural stability, making BTCZH10 the most efficient and pH-tolerant formulation.

### 3.3. Model fitting and statistical analysis

The CCD experimental matrix and the corresponding responses are summarized in Table 3. Each experimental run was performed according to the design matrix, and the PDE was measured as the actual response. The predicted PDE values obtained from the quadratic model showed a good agreement with the experimental data, confirming the reliability of the fitted model. The slight differences between actual and predicted values can be attributed to experimental variability, while the overall trend demonstrates the adequacy of the model in capturing the response behavior across the design space.

CCD results for the photodegradation of BCG by the BTCZH*x* catalysts

**Table:** 

Independent variables	Units	Coded low	Coded high
pH	—	−1 ↔ 3.0	+1 ↔ 11.0
Process time (≡ *t*)	min	−1 ↔ 0.0	+1 ↔ 35.0
Si/Zr molar ratio (≡*x*)	—	−1 ↔ 5.0	+1 ↔ 20.0

**Table:** 

RUN	pH	*t* (min)	*x*	BCG PDE (%)	
				Actual	Predicted
1	11.0	35.0	5.0	8.5	18.2
2	7.0	20.0	12.5	72.3	72.6
3	3.0	5.0	20.0	83.3	78.9
4	11.0	5.0	5.0	1.4	0.0
5	13.7	20.0	12.5	2.5	0.0
6	3.0	35.0	5.0	75.4	68.4
7	11.0	5.0	20.0	3.3	15.6
8	3.0	35.0	20.0	95.8	104.1
9	7.0	20.0	12.5	72.3	72.6
10	7.0	20.0	12.5	72.3	72.6
11	11.0	35.0	20.0	6.9	9.4
12	7.0	20.0	12.5	72.3	72.6
13	7.0	20.0	12.5	72.3	72.6
14	7.0	0.0	12.5	50.0	47.2
15	3.0	5.0	5.0	14.6	17.3
16	7.0	20.0	25.1	57.0	48.5
17	7.0	20.0	0.1	5.0	6.1
18	7.0	20.0	12.5	72.3	72.6
19	7.0	45.2	12.5	80.5	74.8
20	0.3	20.0	12.5	85.0	87.8

The regression analysis yielded the following quadratic polynomial model in terms of coded variables:

2 BCG PDE = −71.12 + 10.23pH + 3.43*t* + 12.80*x* − 0.13pH × *t* − 0.37pH × *x* − 0.06*t* × *x* − 0.72pH^2^ − 0.03 *t*^2^ − 0.29 *x*^2^

The statistical significance of the model was evaluated through ANOVA (Table 4). The quadratic model was found to be highly significant (*p* < 0.0001) with an *F*-value of 38.91. Among the main effects, pH (A) exerted the most dominant influence on PDE (*F* = 174.72, *p* < 0.0001), followed by the Si/Zr ratio (*x*, C) and process time (*t*, B). Interaction terms (AB, AC, and BC) were also significant, indicating that the effect of each factor depends on the levels of the others. Furthermore, the quadratic terms (*A*^2^, *B*^2^, and *C*^2^) were significant, particularly *C*^2^ (*F* = 60.23, *p* < 0.0001), which reflects the curvature in the response surface associated with the Si/Zr molar ratio.

**Table 4:** ANOVA results for the quadratic model

Source	Sum of squares	df	Mean square	*F*-value	*p*-value
Model (significant)	22058.46	9	2450.94	38.91	<0.0001
A-pH	11006.74	1	11006.74	174.72	<0.0001
B-*t*	1536.25	1	1536.25	24.39	0.0006
C-*x*	2364.29	1	2364.29	37.53	0.0001
AB	488.84	1	488.84	7.76	0.0193
AC	988.39	1	988.39	15.69	0.0027
BC	336.10	1	336.10	5.34	0.0435
*A* ^2^	1933.07	1	1933.07	30.69	0.0002
*B* ^2^	377.35	1	377.35	5.99	0.0344
*C* ^2^	3794.38	1	3794.38	60.23	<0.0001
Residual	629.96	10	63.00		
Lack of fit	629.96	5	125.99		
Pure error	0.00	5	0.00		
Cor total	22688.41	19	—		
*R* ^2^	0.97		Std. dev	7.94	
Adjusted *R*^2^	0.95		Mean	50.14	
Predicted *R*^2^	0.75		C. V%	15.83	
Adeq precision	19.92				

The model exhibited strong statistical performance with an *R*^2^ of 0.97 and an adjusted *R*^2^ of 0.95, indicating that 95% of the variability in PDE is explained by the model. The predicted *R*^2^ (0.75) was in reasonable agreement with the adjusted *R*^2^, confirming the predictive power of the model. The standard deviation (7.94) and C.V. (15.83%) were within acceptable limits, while an adequate precision value of 19.92 indicated a strong signal-to-noise ratio. Overall, the CCD results in Table 3 combined with the ANOVA analysis (Table 4) confirm that the quadratic model provides a robust and accurate description of the relationship between pH, process time, and Si/Zr ratio with respect to BCG photodegradation efficiency.

**Fig. 6:**
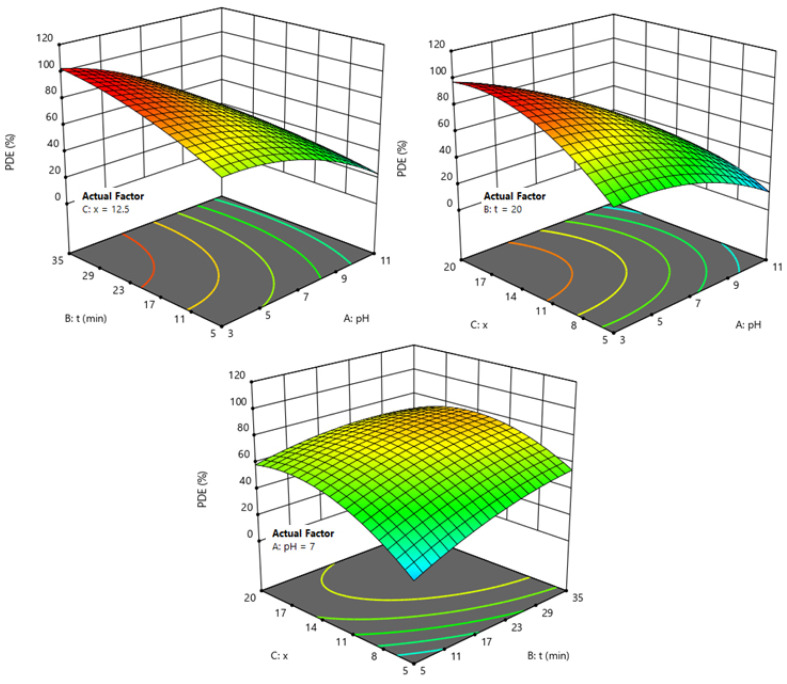
Effects of pH, process time and Si/Zr molar ratios on photodegradation efficiency of BCG.

The three-dimensional response surface plots (Fig. 6) provide a clear visualization of the interactive effects of pH, process time, and Si/Zr molar ratio on the PDE of BCG. Among the studied factors, pH emerged as the most critical parameter. At acidic conditions (pH ≈ 3), PDE reached values above 90%, particularly when combined with higher Si/Zr molar ratios (*x* ≈ 20) and longer irradiation times. This significant enhancement can be attributed to the increased surface protonation of the catalyst, which promotes dye adsorption and improves charge carrier separation, thereby accelerating the photocatalytic process. Conversely, under alkaline conditions (pH ≈ 11), PDE decreased sharply to below 10%, irrespective of process time or molar ratio. This decline is explained by the repulsive interactions between negatively charged catalyst surfaces and anionic dye species at higher pH, which suppresses adsorption and reduces photocatalytic activity. Process time also positively influenced PDE, with extended irradiation (up to 35 min) markedly improving degradation under neutral and acidic conditions, whereas its effect was negligible at alkaline pH. The Si/Zr molar ratio exhibited a similar trend, where higher ratios correlated with improved PDE, suggesting that zirconium incorporation enhances charge transfer and visible-light utilization. These findings highlight the strong interactive effects (pH × *x* and pH × *t*), emphasizing that maximum PDE is achieved only under specific combinations of the three parameters.

**Fig. 7:**
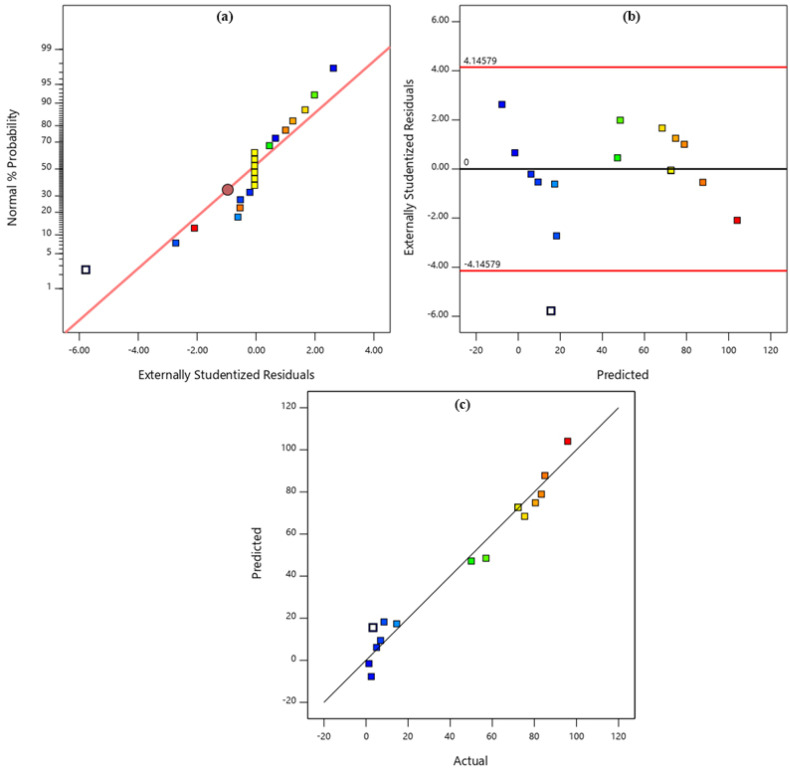
The experimental PDE values plotted against the predicted BCG dye PDE values obtained from the model, (a) normal plot of residuals, (b) residuals *vs.* predicted data, and (c) predicted *vs.* actual data.

The adequacy of the developed quadratic model was further validated through diagnostic plots (Fig. 7). The normal probability plot of residuals (Fig. 7a) indicated that the residuals followed a straight line, confirming the assumption of normal distribution. The plot of residuals *versus* predicted values (Fig. 7b) showed random scattering around zero without systematic trends, verifying the independence and homoscedasticity of the errors. Finally, the correlation between predicted and actual values (Fig. 7c) demonstrated a strong linear relationship, with most data points closely aligned along the diagonal line. This excellent agreement confirms the robustness and reliability of the model in predicting the experimental outcomes. Collectively, the response surface plots and diagnostic analyses demonstrate that the developed quadratic model not only provides valuable insights into the interactions among the studied parameters but also accurately predicts the photodegradation efficiency of BCG under a wide range of experimental conditions.

To explore the impact of variables on the experimental response, the experimental design was implemented to identify optimal conditions. Given that achieving the maximum PDE is the primary goal for this photocatalytic degradation process, the optimal conditions—resulting in 97.85% efficiency—were identified as a pH of 4.60, a processing time of 32.90 min and Si/Zr molar ratio of 16.41.

### 3.4. Stability test and the effect of visible light

**Fig. 8:**
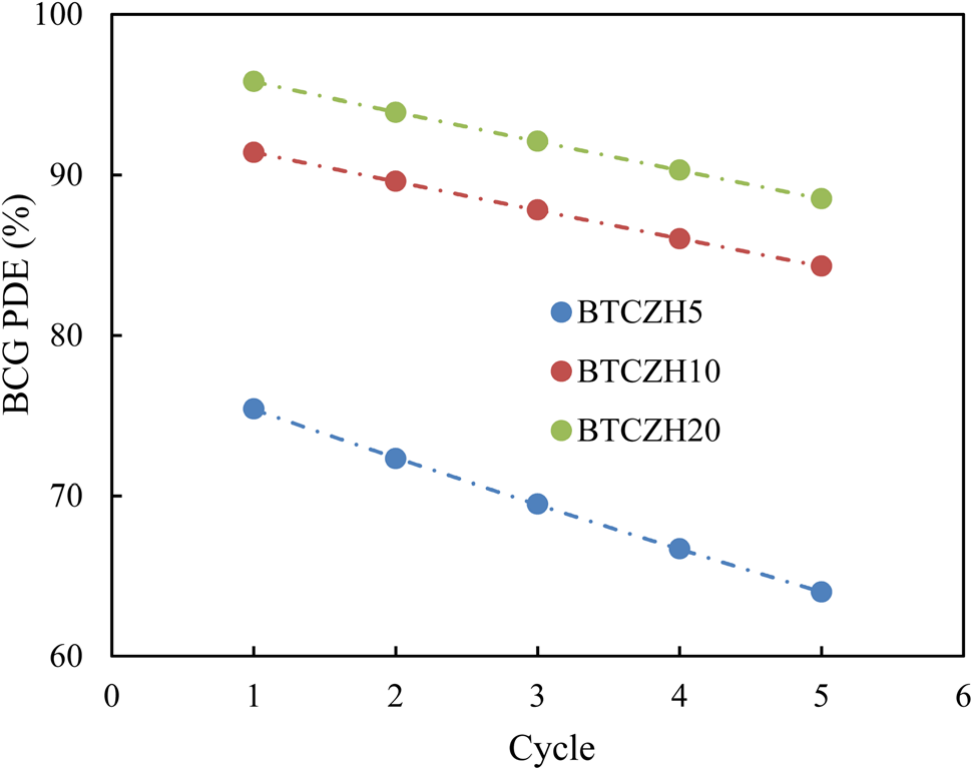
Stability and reusability of BTCZH*x* photocatalysts over five successive cycles for BCG photodegradation under optimal conditions (pH = 3, *t* = 35 min, visible light irradiation).

The stability and reusability test results (Fig. 8) demonstrate the long-term performance of the BTCZH*x* photocatalysts. All three catalysts maintained significant photocatalytic activity over five consecutive runs, indicating good structural stability under the applied reaction conditions.

However, a gradual decrease in PDE was observed, which can be attributed to several factors, including partial loss of active sites during the recovery process, adsorption of intermediate organic species on the catalyst surface, and minor structural changes upon repeated use. Among the studied catalysts, BTCZH20 exhibited the highest stability, retaining nearly 90% of its initial activity after five cycles. BTCZH10 also showed good reusability, with only a slight decrease (∼7%) in efficiency across cycles. In contrast, BTCZH5 experienced a more pronounced decline, losing about 15% of its initial PDE by the fifth run. These results suggest that increasing the Si/Zr molar ratio enhances both the photocatalytic activity and the durability of the catalysts. The excellent recyclability of BTCZH20 in particular highlights its potential for practical applications in wastewater treatment, where long-term catalyst stability is crucial.

### 3.5. Proposed mechanism of BCG dye photocatalytic degradation

The photocatalytic degradation of BCG dye over the BTCZH*x* catalysts can be explained by a mechanism involving light-induced charge carrier generation, surface redox reactions, and the formation of reactive oxygen species (ROS).^21,30^ Upon visible-light irradiation, the catalyst absorbs photons and generates electron–hole pairs (eqn (3)). The incorporation of Zr into the Si framework improves charge separation and facilitates electron transfer, as evidenced by the enhanced photodegradation efficiency observed at higher Si/Zr ratios (Table 3 and Fig. 6).

3 BTCZH + *hv* → *e*_CB_^−^ + *h*_VB_^+^

The photogenerated electrons in the conduction band (CB) react with dissolved oxygen molecules to produce superoxide radicals (˙O_2_^−^), while the photogenerated holes in the valence band (VB) oxidize water or surface hydroxyl groups to yield hydroxyl radicals (˙OH),^31^ as shown in eqn (4)–(6). Both ˙OH and ˙O_2_^−^ are highly reactive oxidizing species responsible for dye molecule attack.

4 O_2_ + *e*_CB_^−^ → O_2_^−^

5 *h*_VB_^+^ + H_2_O → OH + H^+^

6 *h*_VB_^+^ + OH^−^ → OH

The formed radicals subsequently degrade the BCG dye molecules through stepwise oxidation, leading to the cleavage of chromophoric groups (–N=N– and aromatic rings) and eventually mineralization into CO_2_, H_2_O, and inorganic ions (eqn (7)):

7 BCG dye + (˙OH,⋅O_2_^−^) → CO_2_ + H_2_O + mineral by products

**Scheme 2:**
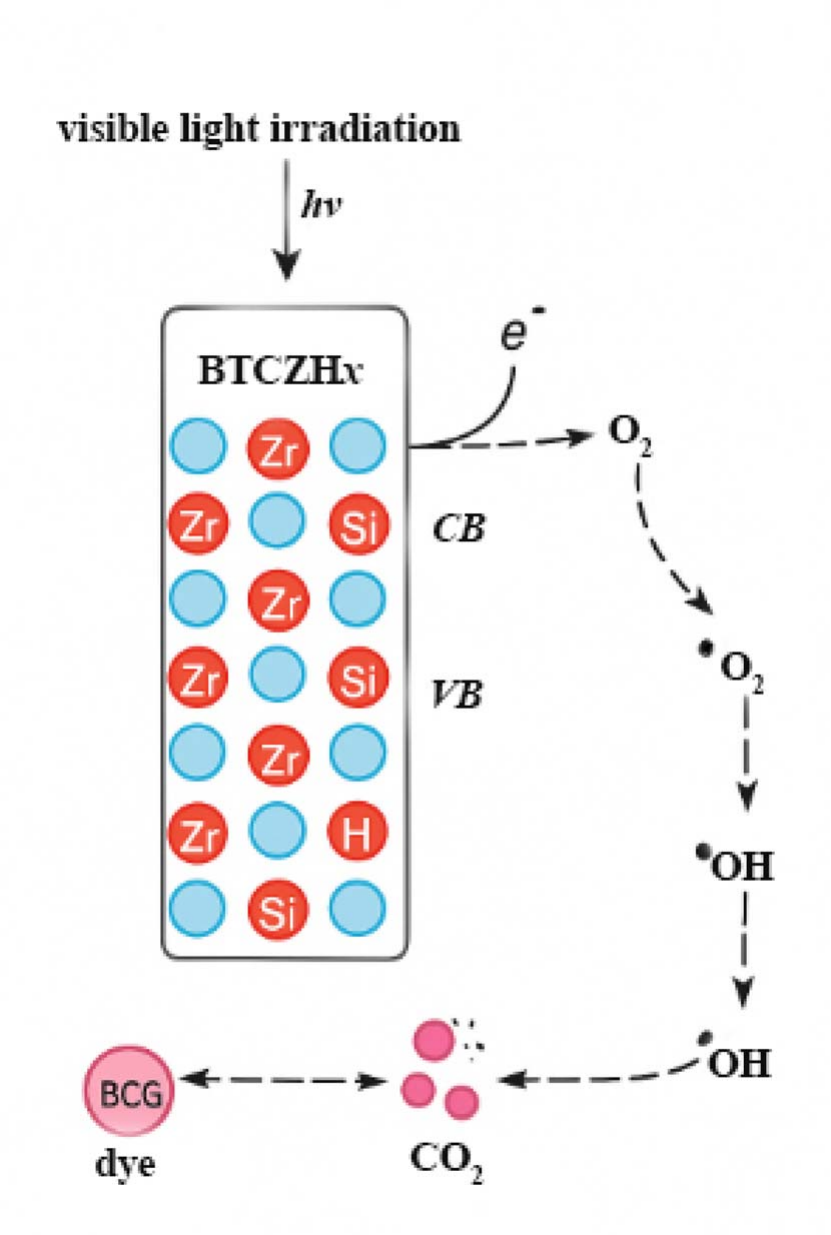
Proposed photocatalytic degradation mechanism of BCG dye over BTCZH*x* catalysts under visible light irradiation.

The influence of pH on the degradation process is consistent with this mechanism. At acidic conditions (pH ≈ 3), the catalyst surface is protonated, which favors the adsorption of the anionic BCG dye molecules and enhances radical attack, resulting in maximum PDE (>90%). In contrast, under alkaline conditions (pH ≈ 11), the catalyst surface becomes negatively charged, leading to electrostatic repulsion with the dye and reduced radical generation efficiency, hence the very low PDE (<10%). Similarly, longer irradiation times promote higher radical accumulation, while optimized Si/Zr ratios enhance charge carrier separation, both of which improve degradation efficiency. A schematic illustration of this proposed mechanism is presented in Schematic 2, showing the sequence of light absorption, charge carrier transfer, ROS generation, and dye degradation. These mechanistic findings are consistent with previous reports on Zr- and Si-modified photocatalysts, which emphasized the synergistic effect of surface acidity, charge separation, and adsorption on photocatalytic efficiency (Scheme 2).^32–34^

The enhanced photocatalytic performance observed at higher Si/Zr ratios can be directly correlated to the catalyst's structural features. Improved charge separation and increased surface acidity favor radical generation and dye adsorption, explaining the observed reactivity trend. This correlation is supported by the data in Table 3 and Fig. 6.

## 4. Conclusion

This study demonstrated the successful synthesis and application of BaTiO_3_/Cr-Zr-HMS (BTCZH*x*) photocatalysts for the efficient degradation of bromocresol green (BCG) dye under visible-light irradiation. Response Surface Methodology (RSM) based on Central Composite Design (CCD) provided a reliable quadratic model (*R*^2^ = 0.97), confirming its predictive capability for photodegradation efficiency (PDE). Among the studied parameters, solution pH was the most critical factor, with acidic conditions (pH = 3) significantly enhancing photocatalytic activity. The optimal operating conditions (pH 3, process time 35 min, Si/Zr molar ratio 20) resulted in a maximum PDE above 95%, validating the robustness of the model predictions. Mechanistic insights revealed that visible-light-induced electron–hole pair generation, followed by the formation of hydroxyl (˙OH) and superoxide (˙O_2_^−^) radicals, played a central role in dye mineralization. Furthermore, reusability tests confirmed the high stability of BTCZH20, which retained nearly 90% of its initial activity after five successive cycles. Overall, the findings highlight BaTiO_3_/Cr-Zr-HMS as a promising and durable photocatalyst for wastewater remediation under visible-light conditions, offering both high efficiency and long-term stability. Future studies may focus on scaling up the process and evaluating its effectiveness in real industrial effluents.

## Author contributions

Study conception and design, material preparation, analysis/investigation, writing—original draft and manuscript—review & editing were done with Dr Nastaran Parsafard. The second author, Dr Ali Akbar Asgharinezhad contributed to writing, review and editing specific sections of the manuscript. Data collection for photocatalytic test was done with Najmeh Karami. I would like to note that the contribution of third author to this work was relatively minor, accounting for less than 1% of the overall research work.

## Conflicts of interest

The authors have no relevant financial or non-financial interests to disclose.

## Supplementary Material

RA-015-D5RA07033C-s001

## Data Availability

All data generated or analyzed during this study are included in this published article. Other data that support the findings of this study are available on request from the corresponding author. Supplementary information (SI) is available. See DOI: https://doi.org/10.1039/d5ra07033c.
