# Supplementary material for: Ferroelectric-assisted BaTiO3/Cr-Zr-HMS (5, 10 & 20) catalysts for efficient visible-light removal of bromocresol green: structure–activity relationship and process optimization
Source: RSC Adv. 2025 Dec 16;15(58):50507–20. doi: 10.1039/d5ra07033c (PMC12706844; doi:10.1039/d5ra07033c)
Supplement: RA-015-D5RA07033C-s001 [file RA-015-D5RA07033C-s001.pdf]

Supplementary Information (SI) for RSC Advances.
This journal is © The Royal Society of Chemistry 2025

Electronic Supplementary Material:

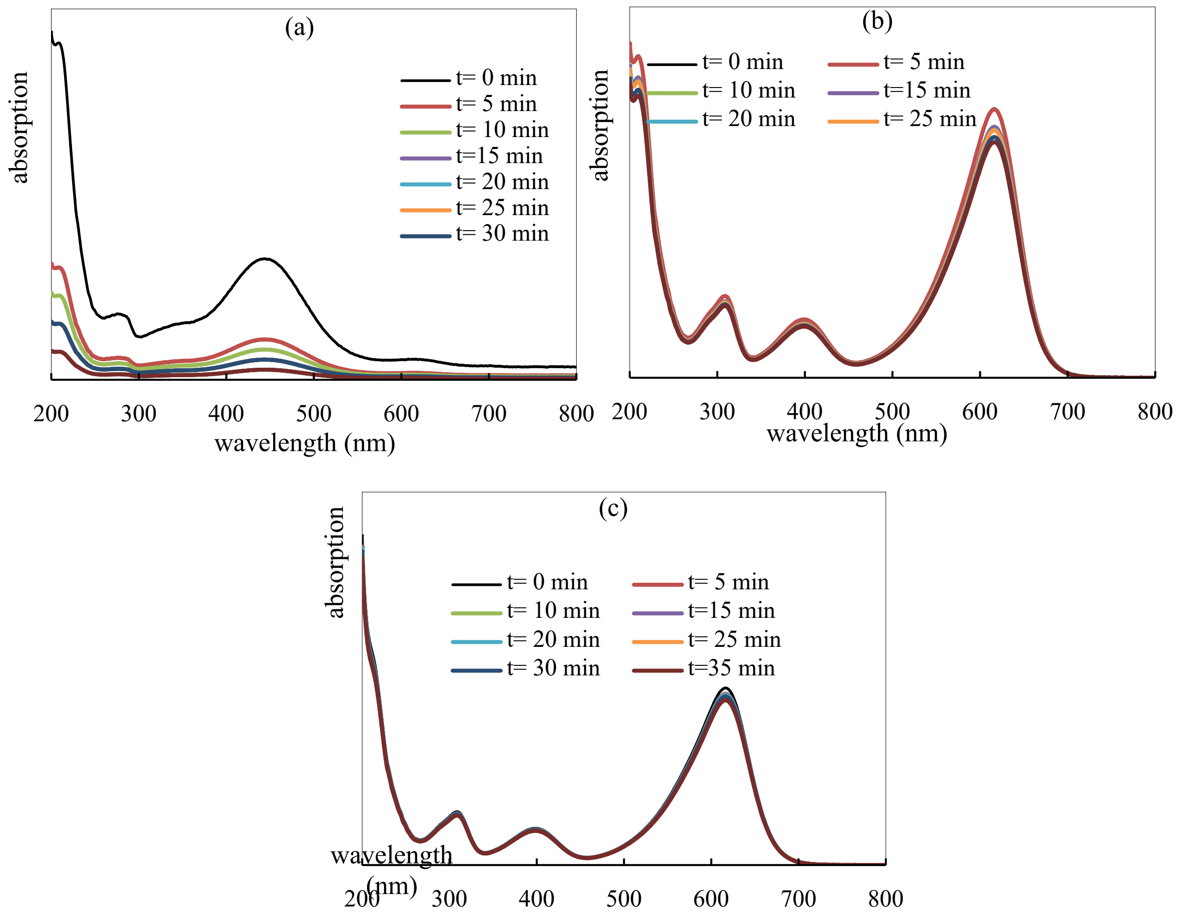

Fig. S1. Representative UV–Vis spectra of BCG dye during photocatalytic degradation at (a) pH 3, (b) pH 7, and (c) pH 11.

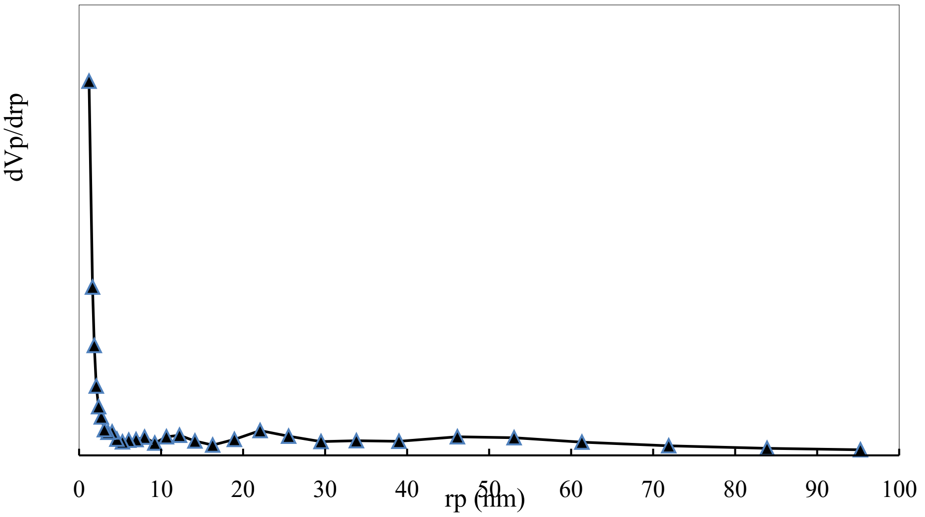

Fig. S2. BJH pore size distribution: pore volume (dV_p/dr_p) vs. pore diameter (nm) for BTCZH*x* catalyst.

Table S1. Mean photocatalytic degradation efficiencies (± standard deviation) of BCG over BTCZH5, BTCZH10, and BTCZH20 catalysts at different pH values, calculated from three independent experimental runs.

t (min)	BTCZH5	BTCZH10	BTCZH20
		pH=3	
5	14.57±0.51	86.13±3.01	83.33±2.92
10	29.65±1.04	86.72±3.04	87.50±3.06
15	38.19±1.34	87.30±3.06	91.67±3.21
20	51.26±1.79	89.45±3.13	91.67±3.21
25	51.76±1.81	89.65±3.14	91.67±3.21
30	71.86±2.52	91.41±3.20	91.67±3.21
35	75.38±2.64	91.41±3.20	95.83±3.35
		pH=7	
5	7.64±0.27	30.52±1.07	0.29±0.01
10	29.36±1.03	50.70±1.77	6.73±0.24
15	45.32±1.59	61.04±2.14	6.88±0.24
20	68.67±2.40	72.26±2.53	7.88±0.28
25	88.92±3.11	84.01±2.94	8.02±0.28
30	97.54±3.41	98.78±3.46	10.60±0.37
35	98.52±3.45	99.48±3.48	12.46±0.44
		pH=11	
5	1.38±0.05	3.99±0.14	3.31±0.12
10	2.08±0.07	4.15±0.14	3.76±0.13
15	3.23±0.11	4.31±0.15	3.76±0.13
20	4.16±0.15	4.46±0.16	3.91±0.14
25	5.31±0.19	5.42±0.19	4.81±0.17
30	5.54±0.19	5.58±0.20	4.81±0.17
35	8.54±0.30	6.22±0.22	6.92±0.24
